# Sanguinarine Regulates Tumor-Associated Macrophages to Prevent Lung Cancer Angiogenesis Through the WNT/β-Catenin Pathway

**DOI:** 10.3389/fonc.2022.732860

**Published:** 2022-06-30

**Authors:** Yajing Cui, Yingbin Luo, Qiaohong Qian, Jianhui Tian, Zhihong Fang, Xi Wang, Yaoying Zeng, Jianchun Wu, Yan Li

**Affiliations:** ^1^ Department of Oncology, Shanghai Municipal Hospital of Traditional Chinese Medicine, Shanghai University of Traditional Chinese Medicine, Shanghai, China; ^2^ Department of Integrated Traditional Chinese and Western Medicine, Obstetrics and Gynecology Hospital, Fudan University, Shanghai, China

**Keywords:** lung cancer, angiogenesis, tumor associated macrophages, sanguinarine, Wnt/β- catenin

## Abstract

Tumor-associated macrophage (TAM)-mediated angiogenesis in the tumor microenvironment is a prerequisite for lung cancer growth and metastasis. Therefore, targeting TAMs, which block angiogenesis, is expected to be a breakthrough in controlling the growth and metastasis of lung cancer. In this study, we found that Sanguinarine (Sang) inhibits tumor growth and tumor angiogenesis of subcutaneously transplanted tumors in Lewis lung cancer mice. Furthermore, Sanguinarine inhibited the proliferation, migration, and lumen formation of HUVECs and the expression of CD31 and VEGF by regulating the polarization of M2 macrophages *in vitro*. However, the inhibitory effect of Sanguinarine on angiogenesis remained *in vivo* despite the clearance of macrophages using small molecule drugs. Further high-throughput sequencing suggested that WNT/β-Catenin signaling might represent the underlying mechanism of the beneficial effects of Sanguinarine. Finally, the β-Catenin activator SKL2001 antagonized the effect of Sanguinarine, indicating that Sanguinarine can regulate M2-mediated angiogenesis through the WNT/β-Catenin pathway. In conclusion, this study presents the first findings that Sanguinarine can function as a novel regulator of the WNT/β-Catenin pathway to modulate the M2 macrophage polarization and inhibit angiogenesis, which has potential application value in immunotherapy and antiangiogenic therapy for lung cancer.

## Introduction

Primary bronchogenic carcinoma (lung cancer) represents the leading cause of cancer death, accounting for 18% of total cancer deaths (1), and is a public health problem worldwide. Tumoral angiogenesis is a prerequisite for the progression, invasion, and metastasis of solid tumors (2). When the cancer is more significant than 1 to 2 mm, new blood vessels will be formed in the microenvironment (3) to transport nutrients and oxygen to tumor cells and clear metabolites. Lung cancer is a highly vascularized tumor (4) and refers to a typical vascular-dependent lesion (5), which is related to its poor prognosis. Therefore, anti-angiogenesis is one of the most critical strategies in the treatment of lung cancer. Currently, the clinical efficacy of traditional antiangiogenic agents alone is not ideal. Consequently, it is of great significance to deeply study the mechanism of angiogenesis.

Researchers have found that macrophage infiltration into the tumor microenvironment, namely, tumor-associated macrophages (TAMs) are usually considered to be the key members of the tumor microenvironment (TME) that is correlated with tumor progression (6, 7). A retrospective study involving patients with lung cancer showed that the number of TAMs in tumor nests is more than 50%, which is much higher than the proportion of other cells such as CD3^+^T cells (8). Accumulating evidence has shown that TAMs trigger “angiogenic switch” (9, 10)by secreting a variety of vascular growth factors (VEGF, Ang2, Tie2, etc.) (11), and matrix metalloproteinases (MMPs) (12), as well as inflammatory factors (TNF-α (13), IL-8 (14), etc.), ultimately resulting in tumor invasion and metastasis. In addition, TAMs are also the cause of treatment failure in chemotherapy (15), radiotherapy, and immunotherapy (16) and are a crucial factor of poor prognoses for lung cancer patients (6). As such, targeting TAMs has gradually become a hot spot at the forefront of tumor research.

Macrophages are characterized by strong heterogeneity and plasticity. M1 type macrophages are activated by lipopolysaccharides (LPS) and the pro-inflammatory cytokine IFN-γ. They exert robust killing activity against pathogens and tumor cells (17) and the promotion of polarized Th1 immune responses by the up-regulation of pro-inflammatory cytokines(TNF-α, IL-1, and IL-6), reactive oxygen species (ROS), and nitric oxide (NO). In contrast, the M2 type is activated by cytokines such as IL-4 or IL-13 and specifically expresses arginase-1 (Arg-1), macrophage mannose receptor (CD206) and YM1 (18), with functions such as inducing the Th2 immune response. Research suggests that M2 macrophages tend to be activated in the tumor microenvironment, and the number of TAMs infiltrating tumor tissues is often positively correlated with poor prognosis in patients. As a result, if the phenotypic transformation of macrophages can be regulated effectively, namely, inhibiting the M2 type and inducing the M1 type, tumor angiogenesis mediated by TAMs can be blocked and the antitumor immune response of the body can be reversed (19).

Sanguinarine, a type of benzophenanthridine alkaloid, possesses various effects, including antibacterial (20), reduce pain (21), and anticancer activities. Current research proves that Sanguinarine has demonstrated significant anticancer effects in a wide variety of tumor types via multiple targets-multiple pathways. Sanguinarine induced apoptosis in pancreatic cancer through the expression Bcl-2 protein levels (22). And, Sanguinarine suppresses the proliferation and migration of gastric cancer cells through the DUSP4/ERK/MMP-9 pathway (23). In hepatocellular carcinoma,Sanguinarine inhibits epithelial-mesenchymal transition(EMT) *via* targeting HIF-1α/TGF-β feed-forward loop (24). In addition, Sanguinarine could inhibit VEGF-mediated angiogenesis through the Akt pathway (25).In summary, Sanguinarine can play an anticancer role through a variety of methods, including inhibiting proliferation and invasion and inducing apoptosis and antiangiogenesis, representing a promising anticancer natural compound with great potential.

However, whether Sanguinarine inhibits angiogenesis through the regulation of macrophages and thereby exerts an anti-lung cancer effect has not yet been reported. In this study, we show for the first time that Sanguinarine can target the WNT/β-Catenin pathway to inhibit the M2 polarization of TAMs and exert antiangiogenic effects on lung cancer and the regulation of immune factors. This finding suggests that Sanguinarine is a potent immunomodulatory agent and angiogenesis inhibitor and has a broad potential in anti-lung cancer applications.

## Materials and Methods

### Cell Culture

The Lewis murine lung adenocarcinoma (Lewis lung cancer cells, LLCs) cell strain and human umbilical vein endothelial cell (HUVEC) strain were both purchased from the Shanghai Cell Bank of Chinese Academy of Sciences; DMEM cell culture medium (KGI Biotechnology Co., Ltd., Jiangsu) with 10% FBS (Gibco, USA) was used to culture the cells in a 37°C incubator.

Selleck Chemicals Co. supplied the Sanguinarine (Catalog: S9032, CAS: 2447-54-3, Chemical Formula: C_20_H_14_NO_4_, Molecular Weight: 332.33, Smiles Structural Formula: C[N+]1=C2C(=C3C=CC4=C(C3=C1)OCO4)C=CC5=CC6=C(C=C52)OCO6). DMSO was used to dilute the Sang into a 10 mM stock solution, which was stored in a -20 refrigerator.

### Subcutaneous Transplanted Tumor Model of Lewis Lung Cancer Mice and Macrophage Clearance

The Animal Ethics Committee of Shanghai Traditional Chinese Medicine Hospital approved all the experimental steps. Six-week-old male C57BL/6 mice (20 ± 2 g) were purchased from Shanghai Slack Laboratory Animal Co., Ltd. and were raised in an SPF environment. Each animal was anesthetized with an intraperitoneal injection of sodium pentobarbital (50 mg/kg).Under the right scapula of each mouse, 5×10^5^ LLCs were injected subcutaneously. Mice began to receive treatment on the next day (day1) with an intraperitoneal injection of normal saline (0.1 ml/mouse/day), Sanguinarine (2.5 mg/kg/day, 5 mg/kg/day) (26) or Cisplatin (DDP) (2 mg/kg, twice a week).

For the model of subcutaneously transplanted tumors with macrophage clearance in Lewis lung cancer mice, was performed as previously published (27). First, on the day before the transplantation of subcutaneous tumors (day -1), mice were injected intraperitoneally with 200 µl of clodronate liposomes (CLPs; US FormuMax, cat: F70101C-AL) for the exhaustion of mononuclear macrophages in mice *in vivo*. Hereafter, CLPs were injected intraperitoneally into mice twice a week, 100 µl per mouse, to continuously exhaust the mononuclear macrophages in mice *in vivo*. At the end of the experiment, the splenic single-cell suspension was extracted. The ratio of CD11b+F4/80+ macrophages was detected by flow cytometry to evaluate the efficiency of macrophage clearance. Mice receive treatment with intraperitoneal injections of normal saline (0.1 ml/mouse/day), Sanguinarine (5 mg/kg/day) or Sanguinarine combined with CLPs. A Vernier caliper was used to measure the size of the tumor every 2 days. Three weeks after administration, the mice were sacrificed and tumor tissues were isolated.

### Preparation for Splenic Single-Cell Suspension

The spleen tissues of mice were extracted and homogenized to form the single-cell suspensions. These suspensions were filtrated through a 70-μm strainer filter, centrifuged at 1000 rpm at 4°C for 5 minutes, and 1 or 2 volumes of red blood cell lysis buffer (Biosharp, China; Catalog: 143191) were added and incubated at room temperature for 5 minutes. Next, 5 ml PBS was added to terminate the lysis. Afterward, the single-cell suspensions were collected and relevant flow antibodies were incubated at 4°C for 30 minutes in the dark. The data was collected with a flow cytometer (Beckman Coulter Inc, USA) and analyzed with FlowJo software (Ashland, OR, USA).

### Paraffin Section Immunostaining

Fresh transplanted tumor tissues in mice were fixed with 4% paraformaldehydeand embedded in paraffin. Tissue sections were stained with anti-mouse CD31 antibody (Bioss, lot: bs-20322R), anti-mouse F4/80 antibody (CST, lot: 30325) and anti-mouse CD206 antibody (CST, lot: 24595). The secondary antibody used in this research was CY3-conjugated AffiniPure goat anti-rabbit IgG (H+L), purchased from Boster (catalog: BA1032). Pictures were taken and recorded using an inverted microscope.

### Extraction of Bone Marrow-Derived Macrophages (BMDMs) in Mice and Construction of an M2 Polarization Model

Five-week-old healthy male C57BL/6 mice were purchased from Shanghai Jie-Si-Jie Laboratory Animal Co., Ltd. The experimental program was as described above (28, 29), where mice were sacrificed by cervical dislocation with adequate cleansing using 75% alcohol, and femora and tibiae of mice were separated, of which marrow cavities were flushed with PBS. The flushing fluid collected was filtered through a 70-mesh screen and centrifuged at 20°C at a rate of 1000 rpm. The supernatant liquid was discarded, and the sediments were resuspended in DMEM containing 10% FBS and M-CSF (Pepro Tech, Lot: #0817245) at a final concentration of 20 ng/ml. The liquid was renewed every 48 h, and the cells were cultured continuously for 5 days. Adherent cells from mice were referred to as bone marrow-derived macrophages (BMDMs). On the 5^th^ day of culture, IL-4 (Pepro Tech, Lot: #021749) at a final concentration of 20 ng/ml was added according to the concentration of 1×10^6^ cells for stimulation for 24 h. Flow cytometry was used to determine the rate of positive expression in M2 macrophages.

### Flow Cytometry and Reagents

FITC anti-mouse CD11b antibody (lot: 101206), PE anti-mouse F4/80 antibody (lot: 12310), and APC anti-mouse CD206 antibody (lot: 141708) were all purchased from BioLegend Co.

After the mature BMDMs were treated under different conditions, the cells were collected under 3% BSA in PBS and blocked at room temperature for 15 minutes. The corresponding flow antibody was added at 4°C and incubated away from light for 30 minutes. The antibody was washed with PBS, and a CytoFLEX flow cytometer (Beckman Coulter Inc, USA) was used for sample detection. FlowJo vX.10 software (Ashland, OR, USA) was used for the analysis of FACS data.

### Cell Counting Kit-8 (CCK-8) Assay

BMDMs well-grown were seeded in a 96-well plate at a density of 3×10^4^ cells per well. The next day, cells were treated with Sang and incubated for 24 h. And then the culture medium was changed with a medium containing 10% CCK-8 solution (YEASEN Biological Company; Shanghai, lot: 40203ES76) and incubated for 1 to 4 h in an incubator. The optical density(OD) value at 450 nm was detected by a microplate reader. Cell viability was calculated in accordance with the formula in the instructions.

### Western Blot

To lyse the cells,RIPA (Beyotime; lot: P0013B) and 1/100 PMSF lysis buffer (Beyotime; lot: ST506) were applied According to the instructions of the BCA quantitative kit (Beyotime; lot: P0010S), the protein content of the samples was measured based on a standard curve of protein. Based on the measured protein content of the sample, 5X loading buffer (Beyotime; lot: P0015) was added(1 μl loading buffer per 4 μl sample)and protein* *samples were* *boiled at 100°C for 8 minutes for protein denaturation. Next, 10% gels (Enzyme Biotechnology, Shanghai, China; lot: PG112) were used for SDS-PAGE analysis. Chemiluminescence Substrate (Thermo Fisher Scientific; lot: 32106) was used for immunoblotting, and finally, the images were analyzed using Quantity One software (BIO-RAD; ChemDoxTM XRS+ with image LabTM software). All antibody information is shown in **Supplementary Table 1**.

### qRT-PCR

Extraction of the total RNA was performed according to the instructions of the TRIzol reagent (Invitrogen), and the RNA was then reverse-transcribed into cDNA by a reverse transcription kit (Takara, Dalian, China). Next, the SYBR Green PCR kit (Takara) was applied to perform real-time PCR according to the manufacturer’s instructions on the QuantStudio 6 Flex real-time PCR system (Thermo Fisher). All the primers were synthesized by Sangon Biotech (Sangon Biotech, Shanghai, China). All the primer sequences used are shown in **Supplementary Table 2**. The 2^-△△Ct^ method was applied to analyze the real-time PCR data (30).

### Preparation of Conditioned Medium (CM)

The experimental method was performed as previously described (31, 32), mature mononuclear macrophages were treated with IL-4, Sang, or the combination treatment for 24 h and then the original medium was replaced by a serum-free medium to be cultured for another 24h.The liquid supernatant was collected and then filtered twice with a 0.45-μM filter and stored in a -80 refrigerator until use. When used, the macrophage-conditioned medium was mixed with the fresh culture medium at a ratio of 1:1, and 10% FBS was added.

### Endothelial Cell Tube Formation Assay

Matrigel (200 µl/well, BD Matrigel matrix, Catalog: 356234) was added to a 24-well plate and incubated at 37°C for 30 minutes for solidification. HUVECs were resuspended in different conditioned media; these cells were inoculated into a 24-well plate at a density of 1.5×10^5^ cells/well and cultured in a 37°C incubator to observe tube formation and to photograph.

### Scratch Wound Healing Assay

HUVECs were seeded into a 6-well plate at a density of 1×10^6^ cells/well; when the cell density reached 80%, 3 parallel scratches was generated in the monolayer with a 200-µl pipette tip. After washing with PBS, the serum-free medium (control group) or macrophage conditioned medium (with or without Sang) was added to the respective wells. Images of the scratch were taken after 0 h, 6 h, 12 h, and 24 h, and the wound area was measured and assessed with the ImageJ software (ImageJ Software Inc., MD, USA).

### Transwell Migration Assay

Transwell inserts (CORNING,lot: 3422) were removed, 50 µl of Matrigel was spread in the upper chamber, and these were placed in a 37°C incubator for 30 minutes. After solidification, 5×10^3^ HUVECs resuspended in serum-free medium were inoculated. Five hundred microliters of complete conditioned medium (containing 10% FBS) was added to the lower chamber; after 12 h-18 h, the upper chamber was removed and wiped off the cells on the surface of the upper chamber. Next, it was fixed with paraformaldehyde (Boster; lot: AR1069) for 30 minutes, stained with crystal violet (Beyotime, lot: C0121-100 ml) for 30 minutes, washed with distilled water twice, repeatedly washed with water and air dried. The cells that migrated through the membranes for each insert were counted under a microscope.

### RNA-Seq Analysis

M0 and M2 cells with good growth status were collected. Three biological replicates were established in each group. After adding TRIzol and blowing repeatedly, they were quickly transferred to an enzyme-free freezing tube. After quick freezing in liquid nitrogen for 30 minutes, they were transferred to a -80°C freezer for long-term preservation; Shanghai Meiji Biotech Co., Ltd. was entrusted to perform eukaryotic mRNA sequencing. Based on the Illumina NovaSeq 6000 sequencing platform, all mRNAs transcribed from cells in each group during the same period were sequenced.

RSEM software package was applied for quantitative statistics of the expression levels of genes and transcripts. Differential gene expression analysis was performed using edgeR and the screening threshold was |log2FC| ≥1 and *p*-value <0.05. Finally, differential genes screened were subjected to pathway enrichment analysis using the KEGG database.

### Statistical Analysis

All the statistical analyses in this article were completed using SPSS software (Version 21.0), and all the data are presented as the mean ± SD of the results of three independent experiments., A t-test was used for statistical analysis for numerical variables conforming to a normal distribution.A One-way analysis of variance (ANOVA) was performed to test the homogeneity of variances between different groups, followed by a *post hoc* LSD test. Statistically significant differences were by the symbols*p<0.05, **p<0.01, and ***p<0.001). Nonsignificant was denoted as “ns” (p>0.05).

## Results

### Sanguinarine-Enhanced Tumor Regression and Angiogenesis Inhibition *In Vivo*

**Figure 1:**
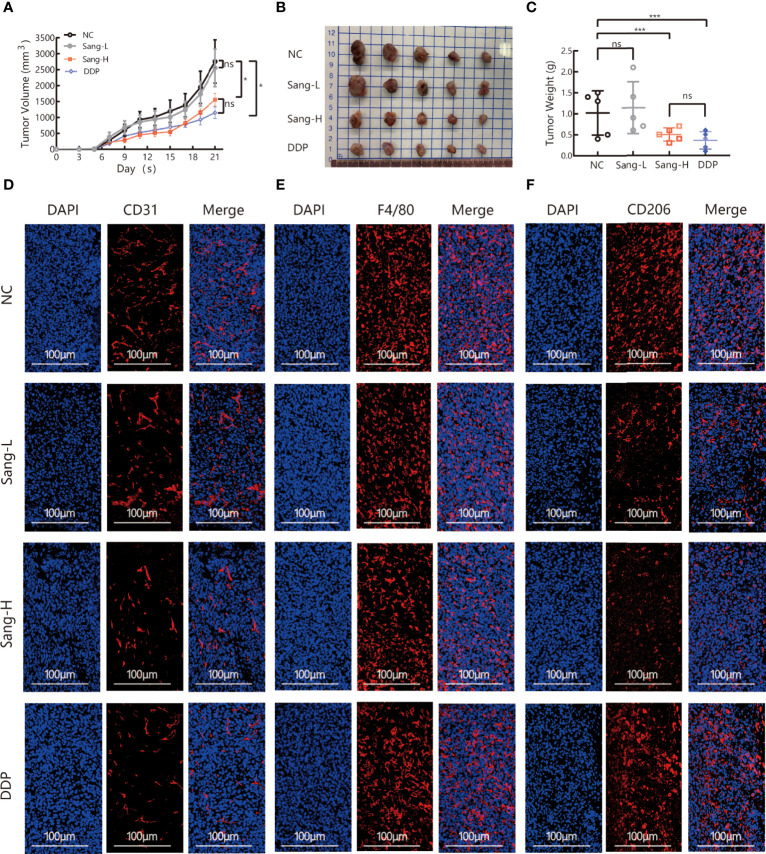
Sang may inhibit tumor growth and angiogenesis in mice through a TAM-dependent mechanism. A total of 5 × 10^5^ LLCs per mouse were subcutaneously injected into the right flank of C57BL/6 mice, and then mice were intraperitoneally injected with saline (0.1 ml/mouse), Sang (2.5 mg/kg, 5 mg/kg) or cisplatin (2 mg/kg) from day 2. Tumor size was measured every *2* days, and following treatment for 3 weeks, the mice were sacrificed, and tumor tissues were isolated. Data are presented as the mean ± SEM of measurements from five mice per group. *p < 0.05; ***p < 0.001. ns, not statistically significant (*p* > 0.*05*). **(A)** Tumor volume curves; **(B)** Tumor tissues collected at the end point; and **(C)** Tumor weights. **(D)** The expression of CD31, **(E)** F4/80, and **(F)** CD206 in the subcutaneous tumor tissue of lung cancer mice were revealed by immunofluorescence staining. Nucleus was stained with DAPI solution. Scale bars: 100, 100, and 100 μm, respectively.

To evaluate the effect of Sanguinarine on tumor growth, C56BL/6 Lewis lung cancer mouse models were used. Different doses of Sanguinarine were injected intraperitoneally (i.p.) for 21 days. The results showed high-dose Sanguinarine and DDP significantly inhibited LLC cell tumor growth (**Figure 1A**). The tumors of mice treated with high-dose Sanguinarine (5 mg/kg) and cisplatin were much smaller (**Figure 1B**) and lighter (**Figure 1C**) compared with mice treated with low-dose Sanguinarine (2.5 mg/kg) or control group.In addition, the body weight of mice was also* *detected. The results showed that mice in the DDP group lost a significant amount of weight, while mice in the low-dose Sanguinarine and high-dose Sanguinarine groups did not have significant weight loss (**Figure S1**), indicating that high-dose Sanguinarine has a better antitumor effect with minor side effects. This was consistent with the results of previous studies (33).

Angiogenesis is necessary for the transport of nutrients and elimination of metabolites for tumor cells. In other words, angiogenesis acts as a bridge for the growth of malignant tumor cells and distant metastasis. CD31, also known as platelet-endothelial cell adhesion molecule (PECAM-1), maintains the migration and survival of endothelial cells and is a direct indicator for the evaluation of angiogenesis (34). To investigate the effect of Sanguinarine on angiogenesis, immunofluorescence was applied to detect the expression of CD31 in the tumor tissues. Compared to the control group, the percentages of CD31+ positive cells were dramatically decreased in the high-dose Sanguinarine group but were unaffected in the low-dose Sanguinarine or the DDP group (**Figures 1D**, **S4A**), indicating that high-dose Sanguinarine can restrain tumor growth and angiogenesis.

Recent research has found that TAMs in the tumor microenvironment induce angiogenesis, which is considered to be the initiating “switch” for tumor angiogenesis and promotes tumor progression. Therefore, we next focused on exploring whether inhibition of tumor angiogenesis by Sanguinarine is related to TAMs from the perspective of the microenvironment. The expression of F4/80, a total macrophage marker, was detected. The results suggested that compared with the control group, there was no dramatic effect on the expression of F4/80 in the Sanguinarine-treated group (**Figures 1E**, **S4B**). Subsequently, the impact of Sanguinarine on CD206, that is, the M2 phenotype, was observed. The results implied that compared with the control group, percentages of CD206+ positive cells (M2-phenotype) were massively reduced in the Sanguinarine group (**Figures 1F**, **S4C**), indicating that the anticancer effects of Sanguinarine may be correlated with the inhibition of angiogenesis and the regulation of M2-type macrophages.

### Inhibition of the M2 Polarization of Macrophages by Sanguinarine

**Figure 2:**
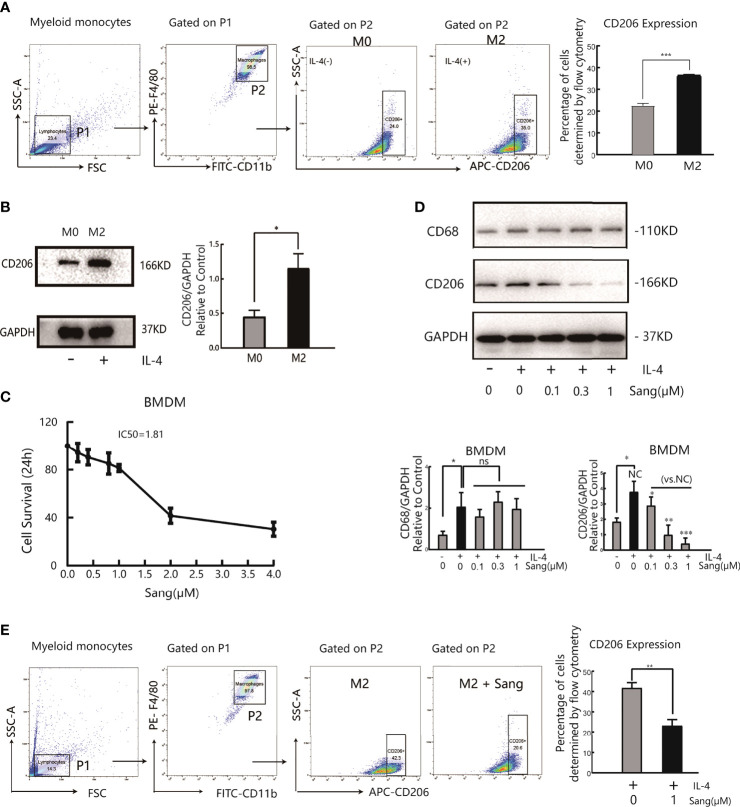
Sang suppresses M2 polarization in macrophages. Bone marrow-derived macrophages (BMDMs) were isolated from mice and treated with IL-4 (20 ng/ml) for 24 h for M2 polarization. **(A)** Flow cytometry analysis of the expression of the M2-like macrophage surface marker CD206. **(B)** Western blot analysis of CD206 protein expression in IL-4- stimulated BMDMs. **(C)** BMDMs were incubated in the presence of Sang at different concentrations (0, 0.2, 0.4, 0.8, 1, 2, and 4 µM) for 24 h, and cell viability was determined by the CCK-8 assay. **(D)** BMDMs in the presence or absence of IL-4 were treated with different concentrations of Sang (0.1, 0.3, and 1 µM), and the expression of CD68 and CD206 proteins was detected through a western blot assay. **(E)** Flow cytometry detection of CD206 expression treated with 1 µM Sang Each experiment was reproduced three times. *p < 0.05; **p < 0.01; ***p < 0.001; ns, not statistically significant (*p* > 0.05).

The results of the above *in vivo* study initially revealed that the antiangiogenic effect of Sanguinarine is related to the regulation of TAMs. Further research on the regulation of Sanguinarine on the TAM phenotype was carried out, based on the different roles of M1 and M2 macrophages in tumor angiogenesis. First, bone marrow-derived macrophages (BMDMs) were isolated and cultured in the presence of M-CSF. After flow cytometry, the purity of CD11b+F4/80+ M0 macrophages was 98%, indicating that they developed into mature macrophages. Then, BMDMs were stimulated with IL-4 to induce M2 polarization. The flow cytometry results displayed that compared with the control group, IL-4 treatment markedly enhanced the CD206+ cell proportion(**Figure 2A**). Moreover, after IL-4 induction, the expression of CD206, an M2-type-specific protein, notably increased (**Figure 2B**). qRT-PCR detected that the mRNA expression of *CD206* and *Arg-1*, both specific markers of M2 macrophages, was increased (**Figure S2B**). In addition, morphological observations suggested that monocytes gradually changed from rounded to flat and spindly under M-CSF culture; the cells became more prolate with pseudopodia after adding IL-4 (morphology of the M2 type) (**Figure S2A**), which is consistent with previous research reports (32). These results suggest that we have successfully established an M2 polarization model of macrophages.

Next, measurements of the IC50 value revealed an IC50 value of 1.81 µM for Sanguinarine-treated BMDMs (**Figure 2C**). Therefore, three types of Sanguinarine concentrations were set for further research that had no obvious effect on BMDM cell proliferation: 0.1 μM, 0.3 μM, and 1μM. The results showed that Sanguinarine markedly suppressed the protein expression of CD206 in M2 macrophages and showed concentration-dependent inhibition, whereas there was no effect on total macrophage CD68 protein expression (**Figure 2D**). The results of flow cytometry suggested that after Sanguinarine intervention, the ratio of F4/80+CD206+ M2 macrophages was 23.23± 5.10%, compared with the untreated F4/80+CD206+ ratio of 41.8 ± 4.47%, with statistical significance (*p*<0.01) (**Figure 2E**). Similarly, morphological alterations were observed under the microscope, and macrophages after Sanguinarine treatment became shorter and rounder with fewer pseudopodia and tended to be similar to the morphology before IL-4 polarization (**Figure S2C**). In summary, Sanguinarine can inhibit the polarization of M2 macrophages, thereby affecting their function.

### Sanguinarine Inhibits Angiogenesis by Restraining M2 Polarization of Macrophages

**Figure 3:**
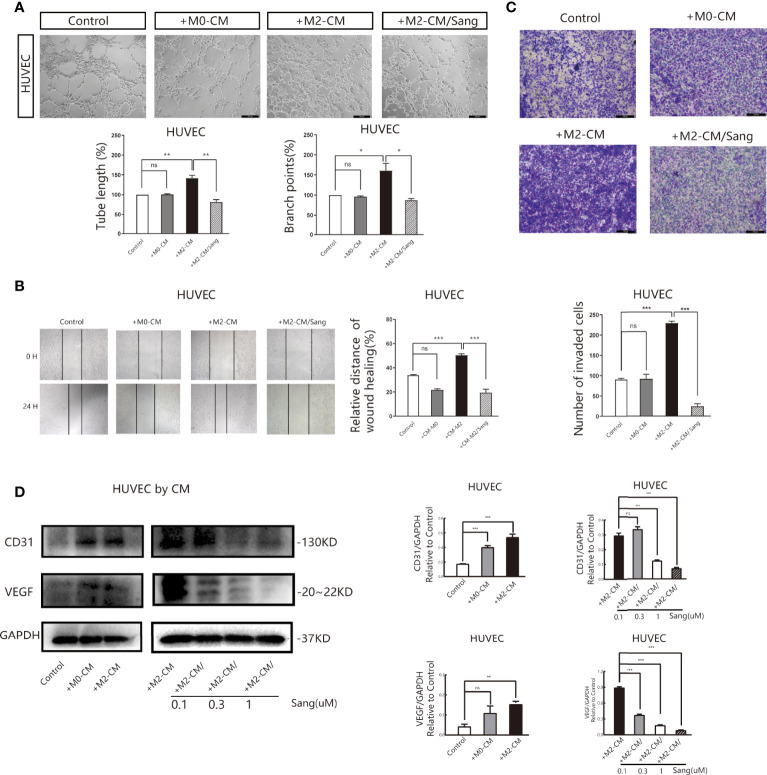
Sang inhibits neovascularization by suppressing M2 macrophage polarization. Conditioned medium (CM) was used to determine the effects of Sang pre-treatment on angiogenesis promoted by M2-like macrophages. **(A)** Representative images of the HUVEC tube formation assay on Matrigel (magnification 20x) and quantification of tubes and branch points. **(B)** Representative images of the HUVECs wound healing assay at 0 h and 24 h, as well as quantitative analysis of the wound healing area. **(C)** HUVECs migration and invasion was detected using a Transwell assay. HUVECs were plated in the upper chamber, and the conditioned medium of macrophages after different treatments was collected and placed in the lower chamber. Representative images of HUVECs invasion, as well as quantification of the number of migrated HUVECs (20X). **(D)** Western blot assay for CD31 and VEGF protein expression in HUVECs that were co-cultured with macrophage-CM, and treated with Sang. Each experiment was reproduced three times. *p < 0.05; **p < 0.01; ***p < 0.001; ns, not statistically significant (p > 0.05).

We also examined whether the inhibition of M2 macrophage polarization through Sang-mediated would affect angiogenesis in the tumor microenvironment. Tube formation Assay revealed that M2-CM could obviously improve the vascular ring density of HUVECs, while most HUVECs in the M2-CM/Sang group did not have ring formation, with a significantly reduced number and density of vascular rings (*p*<0.01) (**Figure 3A**). Proliferation (35, 36) and migration of endothelial cells are both necessary steps for angiogenesis. The Wound Healing Assay results implied that M2-CM was able to increase the wound-healing rate compared to the untreated control, while this trend was reversed with the treatment of Sang (*p*<0.01) (**Figure 3B**). Transwell experiment results indicated that the number of HUVECs in the M2-CM group passing through the chamber increased significantly, while the number of HUVECs in the M2-CM/Sang group was considerably reduced (**Figure 3C**). The results above indicate that Sanguinarine can inhibit angiogenesis through inhibition of the polarization of M2 macrophages.

Vascular endothelial growth factor (VEGF) is the essential inducing factor for proangiogenesis, and when combined with the VEGF receptor (VEGFR) on endothelial cells, it mediates angiogenesis, changes vascular permeability, and mediates inflammation (37). Experimental results indicated that under M2-CM culture, the expression of CD31 and VEGF in HUVECs was critically increased, while under M2-CM/Sang culture, the expression of CD31 and VEGF in HUVECs was significantly inhibited (**Figure 3D**). Overall, these findings suggest that Sanguinarine may affect capillary like-tube formation of endothelial cells by regulating M2-type macrophage polarization.

### Sanguinarine Inhibits Tumor Growth and Angiogenesis in Mice by Regulating the Polarization of M2 Macrophages

**Figure 4:**
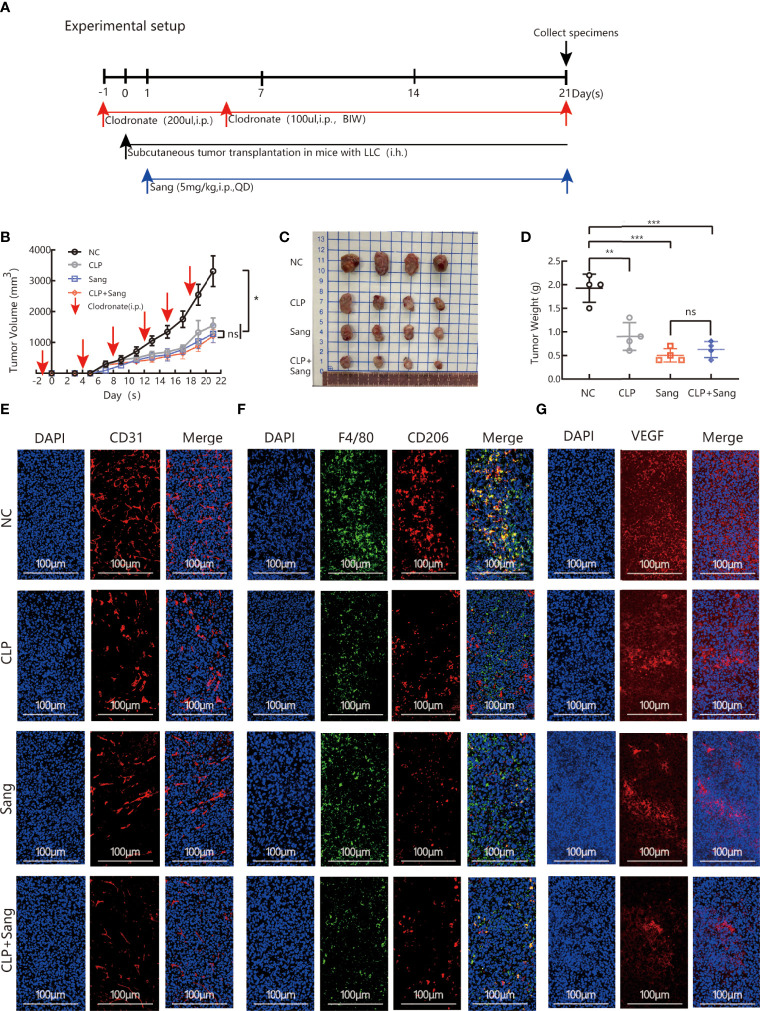
Sang inhibits tumor growth and neovascularization in mice by suppressing macrophage M2 polarization. CLP (200 µl/mouse) was intraperitoneally administered on the day before starting tumor inoculation (day -1). A total of 5 × 105 LLCs per mouse were subcutaneously injected into the right flank of C57BL/6 mice (day 0), and then 100 µl of CLP per mouse was administered by intraperitoneal injection on days 4, 8, 12, 15 and 19 and Sang (5 mg/kg) from day 1. Tumor size was measured every 2 days, and following treatment for 3 weeks, the mice were sacrificed and tumor tissues isolated. Data are presented as the mean ± SEM of measurements from five mice per group. *p < 0.05; **p < 0.01; ***p < 0.001; ns, not statistically significant (p > 0.05). **(A)** Experimental setup for depletion of macrophages in the Lewis lung subcutaneous transplantation mouse model. **(B)** Tumor volume curves; **(C)** Tumor tissues collected at the end point; and **(D)** Tumor weights. **(E)** The expression of CD31, **(F)** F4/80 colocalization with CD206, and **(G)** VEGF in the subcutaneous tumor tissue of lung cancer in macrophage-cleared mice was revealed by immunofluorescence staining. Nucleus was stained with DAPI solution. Scale bars: 100, 100, and 100 μm, respectively. *In vivo*, Sang reduced the secretion of VEGF by inhibiting M2 polarization, which ultimately resulted in inhibition of angiogenesis and tumor growth in mice.

The above studies indicate that Sanguinarine can inhibit angiogenesis by regulating M2 macrophage polarization. Furthermore, our next objective was to evaluate if the findings *in vitro* were consistent with the effects *in vivo*. The model of macrophage clearance in Lewis lung cancer mice was constructed, as shown in the experimental flow chart (**Figure 4A**). The results showed that macrophage clearance alone and Sanguinarine treatment alone could inhibit tumor growth,(**Figure 4B**), manifesting in the smallest tumor (**Figure 4C**) and the lightest tumor (**Figure 4D**). The percentage of CD11b+F4/80+ macrophages significantly decreased in the spleen and tumor tissue treated with macrophage clearance (**Figure S3** and **Figures 4F**, **S6B, E**), which is consistent with previous reports (38–40). In addition, the group treated with CLPs effectively decreased tumor size and load (**Figures 4B–D**), indicating that macrophages contribute to neoplastic progression. Unexpectedly, the tumor inhibition effect in the combination group was not significantly different from that in the Sanguinarine alone group, which could be related to the multitarget characteristics of Sanguinarine.

Immunofluorescence co-staining demonstrated that the combination group had significantly reduced CD206 expression,(**Figures 4F**, **S5B**, **S6A–D**), which suggests that Sanguinarine exerts an influence by regulating the M2 phenotype but not reducing the overall population of macrophages.

Expression of the blood vessel marker CD31 in the tumor mass was detected by immunofluorescence. The results showed that clearance of macrophages reduced the signal expression of CD31. After combination therapy of macrophage clearance and Sanguinarine treatment, there was a more significant reduction in the signal expression of CD31 (as shown in **Figures 4E**, **S5A**). As expected, VEGF expression was decreased when treated with CLP and Sanguinarine alone, whereas the combination treatment exhibited a synergistic suppression (**Figures 4G**, **S5C**). Above all, these results suggest that tumor angiogenesis depends on M2 macrophages, and that Sanguinarine exerts an antiangiogenic effect by reducing VEGF expression, which is related to the regulation of M2 macrophages.

### High-Throughput Screening of Differentially Expressed Genes of Macrophage Polarization Modulated by Sanguinarine

**Figure 5:**
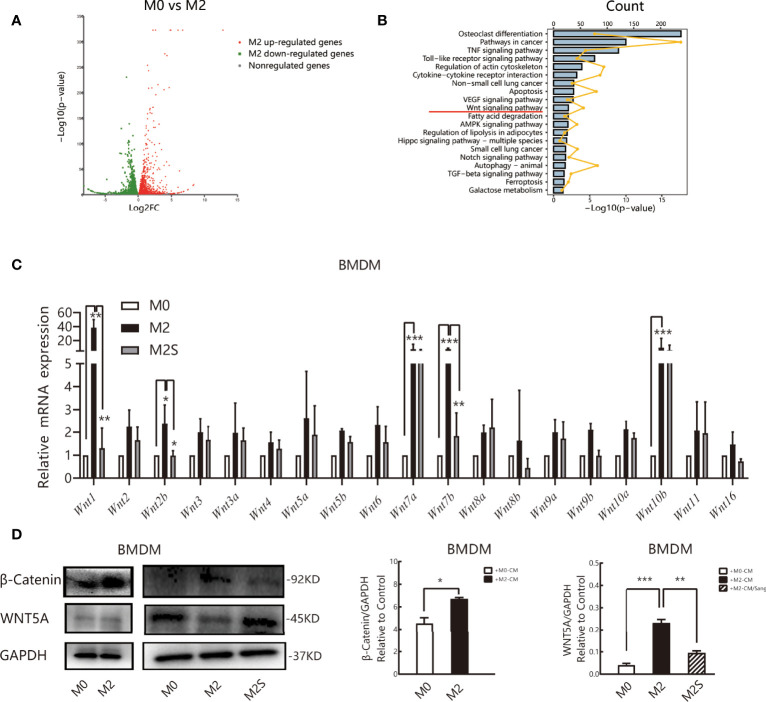
Sang regulates the M2 phenotype of macrophages by the WNT/β-Catenin pathway. RNA sequencing (RNA-Seq) was used to profile genome-wide gene expression and transcriptome changes in M0 and M2 macrophages. |log2FC| >1 and *p*-value<0.05 were taken as thresholds to screen differential genes. **(A)** Volcano plot showing the differentially expressed genes (DEGs) in M0 versus M2, n = 3. FC, fold change. **(B)** KEGG pathway enrichment analysis of differentially expressed genes (DEGs). Each of these blue entries represents a signaling pathway; broken yellow lines indicate the number of differential genes enriched in the pathway. **(C)** qRT-PCR was performed to determine *Wnt* ligand expression in M0, M2, and M2S. **(D)** Western blot assay for WNT5A and β-Catenin in M0, M2, and M2S, are considered to be key proteins in the non-classical and classic WNT/β-Catenin pathway, respectively. *p < 0.05; **p < 0.01; ***p < 0.001.

To clarify the molecular mechanism of Sanguinarine in the regulation of M2, RNA sequencing (RNA-seq) was subsequently applied to detect the whole-genome transcriptome levels of M0 and M2., The screening threshold for the genetic analysis of between-group variance was |log2FC| ≥1 and a *p*-value <0.05. We identified 6462 differential genes in the M2 vs. M0 group, of which 3370 were upregulated and 3092 were downregulated (**Figure 5A**). KEGG pathway analysis identified that these differentially expressed genes are mainly involved in the osteoblast differentiation pathway, cancer pathway, Toll-like receptor signaling pathway, cytokine receptor signaling pathway, VEGF signaling pathway, apoptosis, ferroptosis, WNT, etc. (**Figure 5B**).

Multiple literature reports have shown that the WNT/β-Catenin pathway is involved in the differentiation of M2 macrophages (41–43), and prior studies by our group suggested that Sanguinarine might inhibit WNT/β-Catenin pathway activation (44). Therefore, RT-qPCR was applied to evaluate the mRNA levels of *Wnt* ligands. The results suggested that compared with M0 macrophages, the expression of all *Wnt* ligands increased in M2 macrophages, among which the expression of *Wnt1, Wnt7a, Wnt7b, and Wnt10b* improved the most significantly (*p*<0.05); after Sanguinarine intervention, compared with M2 macrophages, the expression of *Wnt* ligands was reduced, among which the expression of *Wnt1 and Wnt7b* was decreased the most significantly (*p*<0.01) (**Figure 5C**). The WNT ligand will bind to its receptor to activate the classic WNT signaling pathway, namely, WNT/β-Catenin, and β-Catenin represents the key switch for signal transmission. Results indicated that the expression of β-Catenin protein increased critically in M2 macrophages (*p*<0.05), while β-Catenin expression was dramatically attenuated after Sanguinarine intervention (*p*<0.01) (**Figure 5D**). The results above imply that WNT signaling is involved in the polarization of M2 macrophages and that Sanguinarine is highly likely to inhibit the polarization of M2 macrophages through inhibition of the WNT pathway.

### Sanguinarine Targets the WNT/β-Catenin Pathway of Macrophages to Restrain M2 Polarization of Macrophages and Lung Cancer Angiogenesis

**Figure 6:**
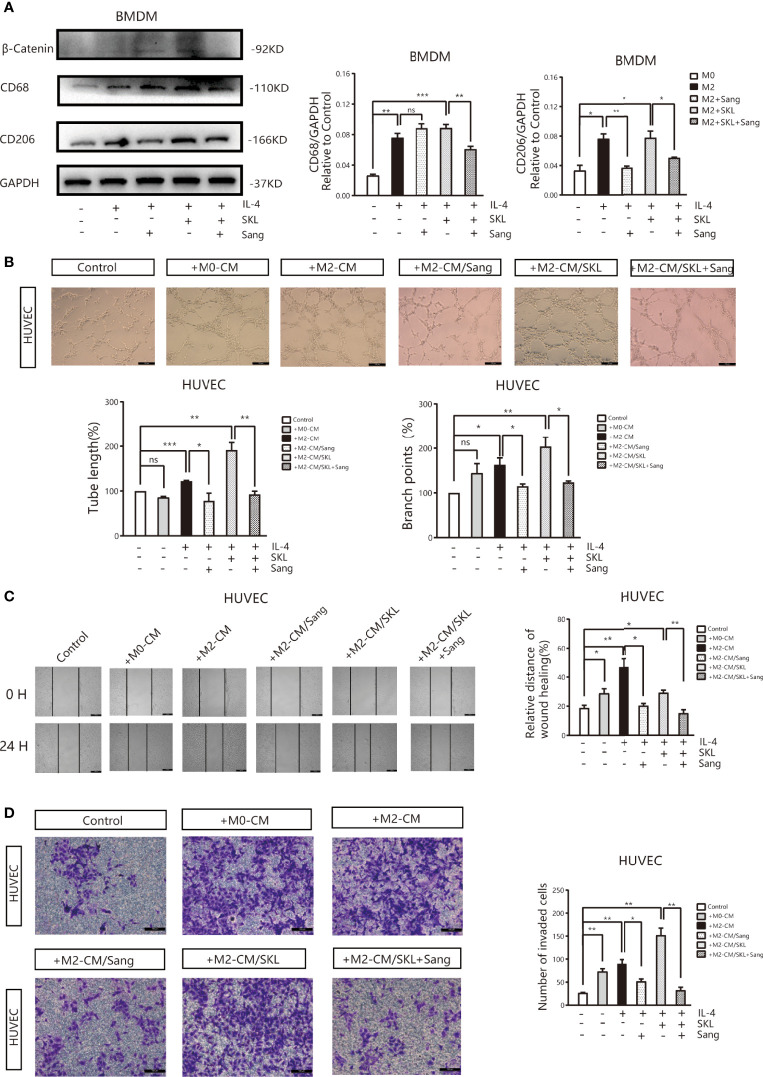
Sang inhibits M2-like polarization of macrophages and M2-mediated angiogenesis by targeting the WNT/β-Catenin pathway in macrophages. **(A)** The WNT/β‐Catenin activator SKL2001, alone or in combined with Sang, was utilized to treat BMDMs. Western blotting was performed to detect the protein expression of CD68, CD206, and β‐Catenin, and the results indicated that SKL2001 could upregulate β-Catenin and CD206 expression; however, β-Catenin and CD206 were markedly decreased following the SKL2001/Sang combination. **(B–D)** HUVECs were cocultured with M2 macrophages’ conditioned medium treated with SKL2001 alone or combined with Sang. **(B)** Representative images of the HUVEC tube formation assay on Matrigel (magnification 20x) and quantification of tubes and branch points. **(C)** Representative images of the wound healing assay of HUVECs at 0 h and 24 h, as well as quantitative analysis of the wound healing area. **(D)** HUVEC migration and invasion was detected using a Transwell assay. HUVECs were plated in the upper chamber, and the conditioned medium of macrophages after different treatments was collected and placed in the lower chamber. Representative images of HUVEC invasion and quantification of the number of migrated HUVECs (20X). Each experiment was reproduced three times. *p < 0.05; **p < 0.01; ***p < 0.001; ns, not statistically significant (*p* > 0.*05*).

To further identify the mechanism of Sanguinarine involving the WNT/β-Catenin signaling pathway, the WNT/β‐Catenin activator SKL2001 was used. The results agreed with the outcome of **Figure ​2C**, showing that the expression of CD206 and β-Catenin was significantly suppressed after Sanguinarine-only treatment of macrophages, while SKL2001-only treatment increased the CD206 and β-Catenin levels. When administered in combination, the efficacy of Sanguinarine was antagonized by SKL2001 (**Figure 6A**).This outcome further proved that the WNT/β-Catenin pathway is involved in M2 polarization and that Sanguinarine can inhibit the WNT/β-Catenin pathway in M2 macrophages as well as the polarization of M2 macrophages.

Moreover, tube formation experiments showed that in HUVECs cultured in SKL2001-CM, the density of vascular rings increased dramatically (*p*>0.05), while in HUVECs cultured in Sanguinarine together with SKL2001-CM, the number and density of vascular rings decreased dramatically (**Figure 6B**). In addition, the scratch experiment showed that scratches were significantly healed in HUVECs treated with SKL2001-CM (*p*<0.01), while in HUVECs cultured with Sanguinarine together with SKL2001-CM, scratch healing was significantly inhibited (*p*<0.01) (**Figure 6C**). In addition, the Transwell experiment showed that HUVEC migration and invasion increased significantly after SKL2001-CM treatment (*p*<0.01), while after treatment with Sanguinarine together with SKL2001-CM, HUVEC migration and invasion were inhibited (*p*<0.01) (**Figure 6D**). In summary, Sanguinarine can inhibit angiogenesis by inhibiting the WNT/β-Catenin pathway of M2 macrophages, which could be proven by the inhibition of the formation of vascular rings, proliferation, and migration of endothelial cells.

## Discussion

Targeting tumor angiogenesis refers to an essential treatment strategy to block the invasion and metastasis of malignant tumor cells and to reduce cancer mortality. In this study, Sanguinarine inhibited the growth of tumors in Lewis lung cancer mice and angiogenesis. The antitumor angiogenesis effect of Sanguinarine is related to the regulation of TAMs, which is not linked to the decrease in total macrophage numbers but to the macrophage phenotype. Further research has found that Sanguinarine could exert an antitumor angiogenic effect by restraining the polarization of M2 macrophages and decreasing VEGF expression in endothelial cells. In addition, Sanguinarine reduced the mRNA expression of *Wnt1 and Wnt7b* and the protein expression of β-Catenin, a nuclear transcription factor in M2 macrophages. The results above suggest that Sanguinarine can exert an antitumor angiogenesis effect by targeting the WNT/β-Catenin pathway of macrophages to inhibit the polarization of M2 macrophages.

Studies have found that tumor angiogenesis has been closely related to the tumor microenvironment in recent years. Hypoxia, the growth factors CSF1 and VEGF, and chemotactic factors in the tumor microenvironment all contribute to increased macrophage recruitment and infiltration into the tumor matrix and blood vessel margins. Research suggests that TAMs are typically M2-polarized immunosuppressive cells in various tumors (45, 46). The M2 TAMs can directly activate angiogenesis through the release of VEGF, bFGF, and PlGF or indirectly activate angiogenesis through the release of matrix metalloproteinases (MMPs) to remodel the extracellular matrix and to improve the migration of endothelial cells (47), which could form new blood vessels and accelerate tumor progression. Therefore, inhibition of the M2 phenotype of macrophages has been proven to be a unique opportunity for effectively blocking tumor angiogenesis (48, 49). The experiments above indicated that inhibition of M2 macrophage polarization could inhibit angiogenesis and VEGF expression. These results are consistent with previous results, which could further confirm that M2-type TAMs promote tumor angiogenesis (31, 50). Previous studies have also found that macrophage clearance therapy alone could not significantly reduce the tumor growth of PDAC homogeneous mice *in situ* (32). However, according to the above findings, Sang and CLP alone significantly inhibited LLC cell tumor growth. Further studies have also shown that the effect of Sanguinarine on tumor suppression is independent of the reduction in the total number of macrophages but is correlated with the inhibition of M2 macrophages. It is considered that it may be due to the sensitivity to CLPs varying between tumor cells and models. In addition, we observed for the first time that the anti-tumor effect was enhanced after treatment by macrophage clearance combined with Sanguinarine. It is believed that TAM phenotypic regulation is not the only target of Sanguinarine. Our previous study pointed to Sanguinarine suppressing lung cancer stem cell (CSC) stemness and inhibiting the proliferation and invasion of lung CSCs (44). In addition, Sanguinarine exhibits multiple antineoplastic characteristics by inhibiting angiogenesis. Therefore, we reasoned that regulating the phenotype of TAMs is only one of the major targets of Sanguinarine action. An apparent synergistic effect is seen when clodronate-liposomes (CLPs) are combined. The results above suggest that Sanguinarine is a promising and exploitable natural anticancer compound with multiple targets and pathways.

The WNT pathway is not only closely related to the invasion and metastasis of lung cancer but also plays a vital role in the maintenance of TAM phenotypes and functions. Therefore, it is a potential clinical molecular target. Previous studies have found (51) that genes from invasive TAMs isolated from transplanted tumors in breast cancer mice were enriched in the WNT signaling pathway. Except for WNT8B, all the other WNT ligands and downstream molecules were increased, with the most obvious ones being WNT7B and 5B. The experiments above indicated that the expression of WNT ligands was significantly increased in M2 macrophages (compared with M0 macrophages), which is consistent with the gene expression analysis of murine myelogenous M2 macrophages in other groups (52). Interestingly, the mRNA levels of *Wnt1, Wnt7a,Wnt7b, and Wnt10b* were higher in M2 macrophages than in M0 macrophages, whereas the expression of *Wnt1 and Wnt7b* dramatically decreased in M2 macrophages after Sanguinarine intervention. These results suggest that macrophages might trigger WNT signaling activation in an autocrine manner to promote the polarization of M2 macrophages. The inhibition of M2 macrophage polarization by Sanguinarine might be related to the inhibition of the WNT pathway.

In addition, the expression of WNT5A in different types of cancers and TAMs remains controversial. In prostate cancer, WNT5A recruits and regulates macrophages through CCL2 to induce castrated prostate cancer (53). In breast cancer, WNT5A from TAMs can induce the expression of MMP-7, resulting in matrix remodeling (54). In contrast, data from our experiments showed that the expression of WNT5A was reduced in M2 macrophages (**Figure 5D**). However, WNT5A has been identified as a promoter protein of the non-classical WNT pathway, induced Ca2+, protein kinase C, Rho-GTPases, and the JNK pathway (55), indicating that the inhibition by Sanguinarine of M2 polarization may depend on the classical WNT/β-Catenin pathway. Among them, β-Catenin represents the key switch for signal transduction to downstream proteins in the classical WNT pathway. The results above showed that the expression of the β-Catenin protein was significantly increased in M2 macrophages, while after Sanguinarine intervention, its expression decreased. Moreover, it was discovered that with SKL2001 intervention, an agonist targeting the β-Catenin signal, the expression of the M2-specific protein CD206 and angiogenesis mediated by M2 increased. However, when the SKL2001 was used for antagonism, the expression of CD206 protein and angiogenesis were reversed, indicating that the WNT signal mediated by TAM-derived β-Catenin plays a central role in the polarization of M2 macrophages, which is consistent with previous research reports (56). Because Sanguinarine attenuated M2 polarization through the WNT/β‐Catenin pathway, it is suggested that Sanguinarine is a new type of regulator for the WNT/β-Catenin pathway targeting the polarization of M2 macrophages, which is considered to be a potential strategy for targeted treatment of lung cancer.

Vascular endothelial growth factor (VEGF), as the most specific angiogenesis-inducing factor, plays an essential role in the initiation of tumor angiogenesis (57). Research shows that WNT7B in breast cancer can stimulate endothelial cells to produce VEGF for angiogenesis (58). It is also observed in the experiments above that VEGF expression on HUVECs was upregulated in coculture with M2-CM compared with monocultured HUVECs. This upregulation was significantly weakened by Sang in HUVECs that were co-cultured with M2-CM (**Figure 3D**). At present, we do not yet know the direct relationship between WNT1, WNT7B, and β-Catenin derived from macrophages and VEGF from endothelial cells. This is also a limitation of our current research and will be the direction and focus of our subsequent research. However, the research above suggests that the WNT signal derived from macrophages may be at least partially involved in tumor angiogenesis. Activation of the WNT pathways is essentially engaged in macrophage-mediated angiogenesis and plays a crucial role in tumor invasion and metastasis.

**Figure 7:**
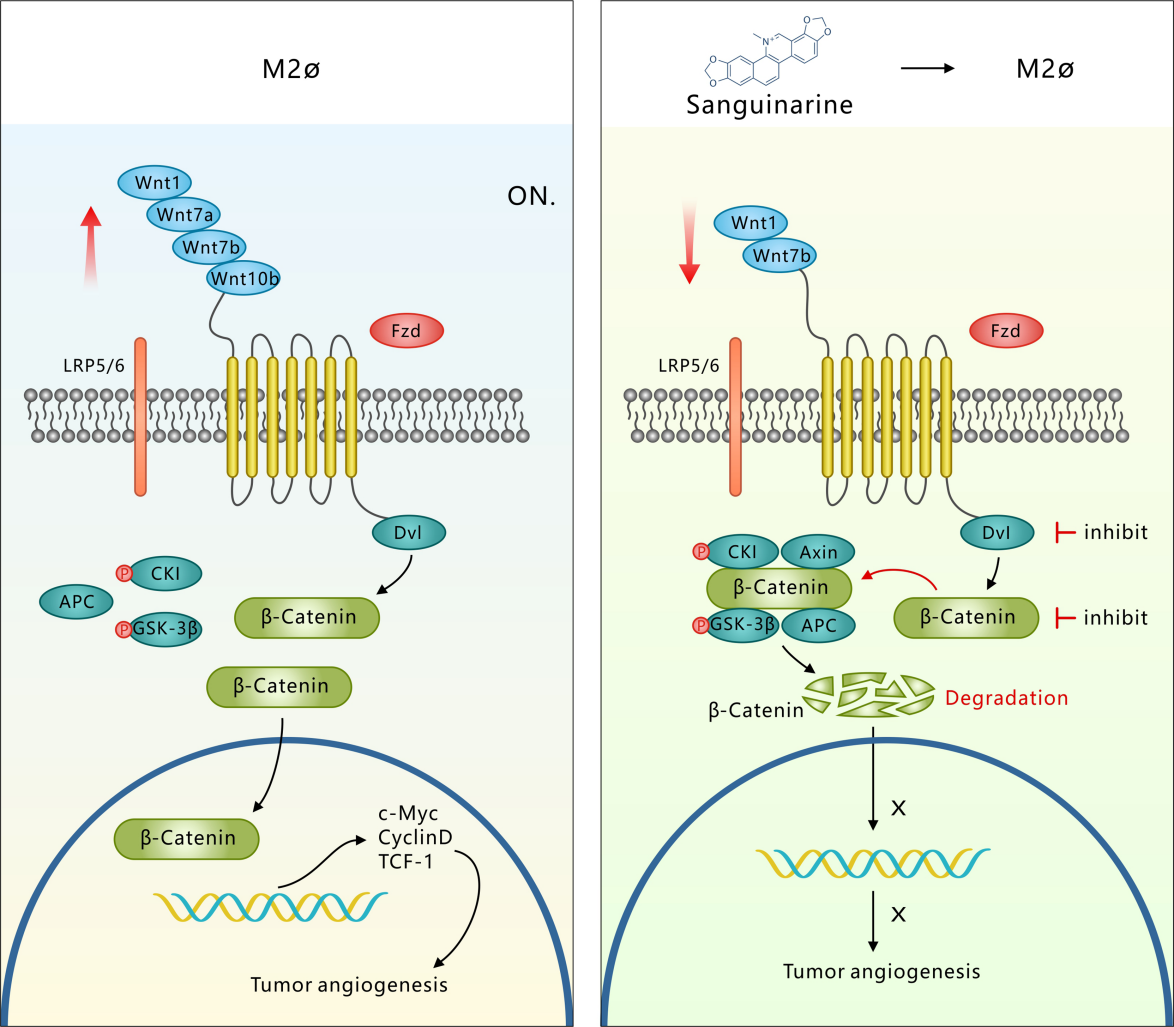
Sang targets the WNT/β-Catenin signaling pathway in TAMs, which represses M2 polarization and thus inhibits TAM-mediated neoangiogenesis. M2-like TAMs show upregulation of WNT ligands (1-7a-7b-10b), leading to transcriptional activation of β-Catenin. β-Catenin translocation into the nucleus induces M2 macrophage polarization, which further promotes the secretion of VEGF protein in HUVECs, leading to the initiation of tumor angiogenesis. However, Sang inhibited WNT/β-Catenin signaling in M2 TAMs by downregulating ligands (*1-7b*) and degrading β-Catenin. β-Catenin could not enter the nucleus to induce M2 macrophage polarization, which thus restrained the secretion of VEGF protein in HUVECs and ultimately suppressed tumor angiogenesis and tumor growth.

Overall, the tumor microenvironment recruits TAMs to the tumor stroma and blood vessel edges through hypoxia and the release of the growth factor CSF1 or chemotactic factors. TAMs, with the abnormally activated WNT pathway and at the same time through autocrine signaling to promote polarization of the M2 type, secrete a variety of pro-angiogenic factors that eventually induce tumor angiogenesis. However, Sanguinarine can inhibit the occurrence of tumor angiogenesis by targeting the WNT/β-Catenin pathway derived from TAMs to inhibit the polarization of M2 macrophages (**Figure 7**). It was discovered for the first time that Sanguinarine is a new regulator for WNT/β-Catenin pathway that targets the polarization of M2 macrophages. This indicates that Sanguinarine is a promising candidate for targeting the polarization of M2 macrophages for antiangiogenic treatment in lung cancer.

## Data Availability Statement

The datasets presented in this study can be found in online repositories. The names of the repository/repositories and accession number(s) can be found below: https://www.ncbi.nlm.nih.gov/ SRP328648, PRJNA745376.

## Ethics Statement

The animal study was reviewed and approved by The Animal Ethics Committee of Shanghai Traditional Chinese Medicine Hospital.

## Author Contributions

YL and JW conceived and supervised the study; YC, JW, and YL designed experiments; YC and YZ performed the animal work; YC, XW, and QQ carried out all *in vitro* experiments; YC, YL, and ZF analyzed data; YL and JT performed the statistical analysis; YL, JW, and YL provided new tools and reagents; YC wrote the original draft and JW reviewed and edited the manuscript; All authors read and approved the final manuscript.

## Funding

This work was financially supported by funds from the National Natural Science Foundation of China (No. 81973795, No. 81603590 and No. 82174183), the Shanghai Pujiang Talent Plan (2020PJD057), the Three-year Action Program of Shanghai Municipality for Strengthening Traditional Chinese Medicine Development (2018–2020) (Grant no. ZY CCCX-4001-01) and the Three-year Action Plan Clinical in Hospital Development Centeritals of Shanghai Shenkang (SHDC2020CR4052).

## Conflict of Interest

The authors declare that the research was conducted in the absence of any commercial or financial relationships that could be construed as a potential conflict of interest.

## Publisher’s Note

All claims expressed in this article are solely those of the authors and do not necessarily represent those of their affiliated organizations, or those of the publisher, the editors and the reviewers. Any product that may be evaluated in this article, or claim that may be made by its manufacturer, is not guaranteed or endorsed by the publisher.
