# Supplementary material for: Sanguinarine Regulates Tumor-Associated Macrophages to Prevent Lung Cancer Angiogenesis Through the WNT/β-Catenin Pathway
Source: Front Oncol. 2022 Jun 30;12:732860. doi: 10.3389/fonc.2022.732860 (PMC9282876; doi:10.3389/fonc.2022.732860)
Supplement: Supplementary file 1 [file DataSheet_1.docx]

**
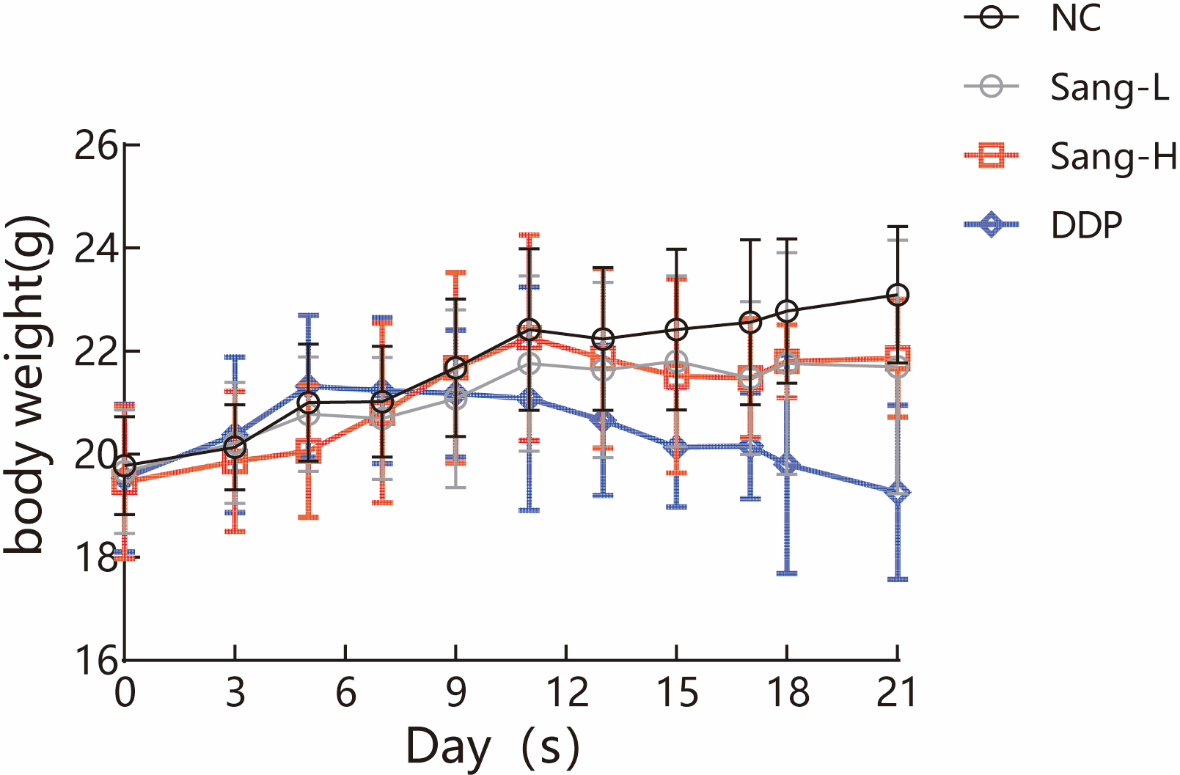
**

**Supplementary Figure 1.** Sang may inhibit tumor growth in mice. Body weight change of mice curve for 21 days.


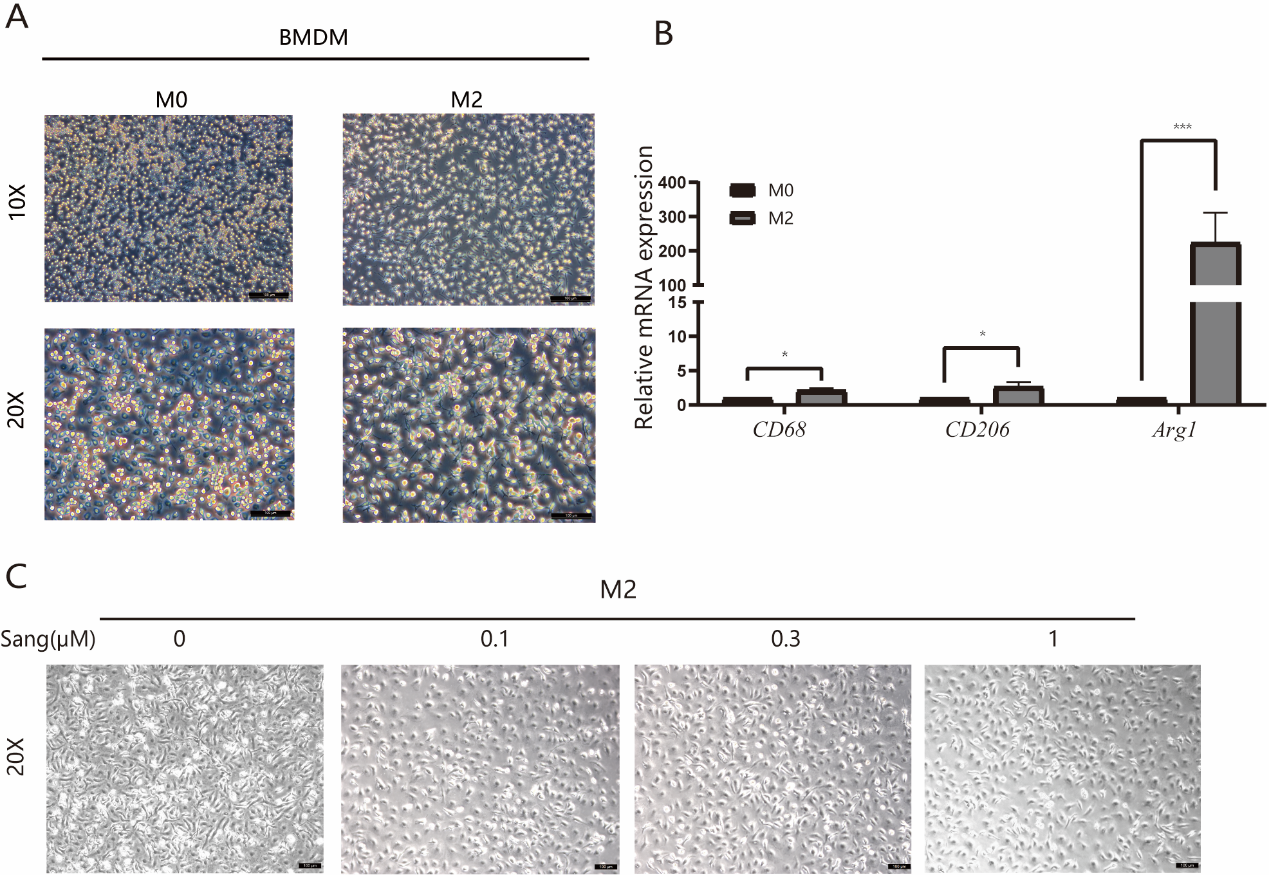


**Supplementary Figure 2.** Sang suppresses M2 polarization in macrophages. (A) Pictures of mature macrophages and polarized macrophages derived from murine bone marrow. Bone marrow-derived macrophages (BMDMs) were isolated and cultured in the presence of M-CSF (10ng/ml) for 5 days to differentiate into mature macrophages (M0 phenotype). Note the changes in the morphology of rounded, loosely adherent monocytes to spindle-shaped and flat appearance M0 macrophages. The M0 macrophages then were stimulated with IL-4 (20ng/ml) for 24h to induce M2 polarization. Cell morphology became more prolate and also developed pseudopod. (B) qRT-PCR detected that the mRNA expression of *CD206* and *Arg-1*, both specific markers of M2 macrophages, was increased. (C) Incubation of M2 macrophages with the Sang resulted in distinct morphological changes, from which cells became shorter and rounder with fewer pseudopodia and tended to be similar to the morphology before IL-4 polarization.


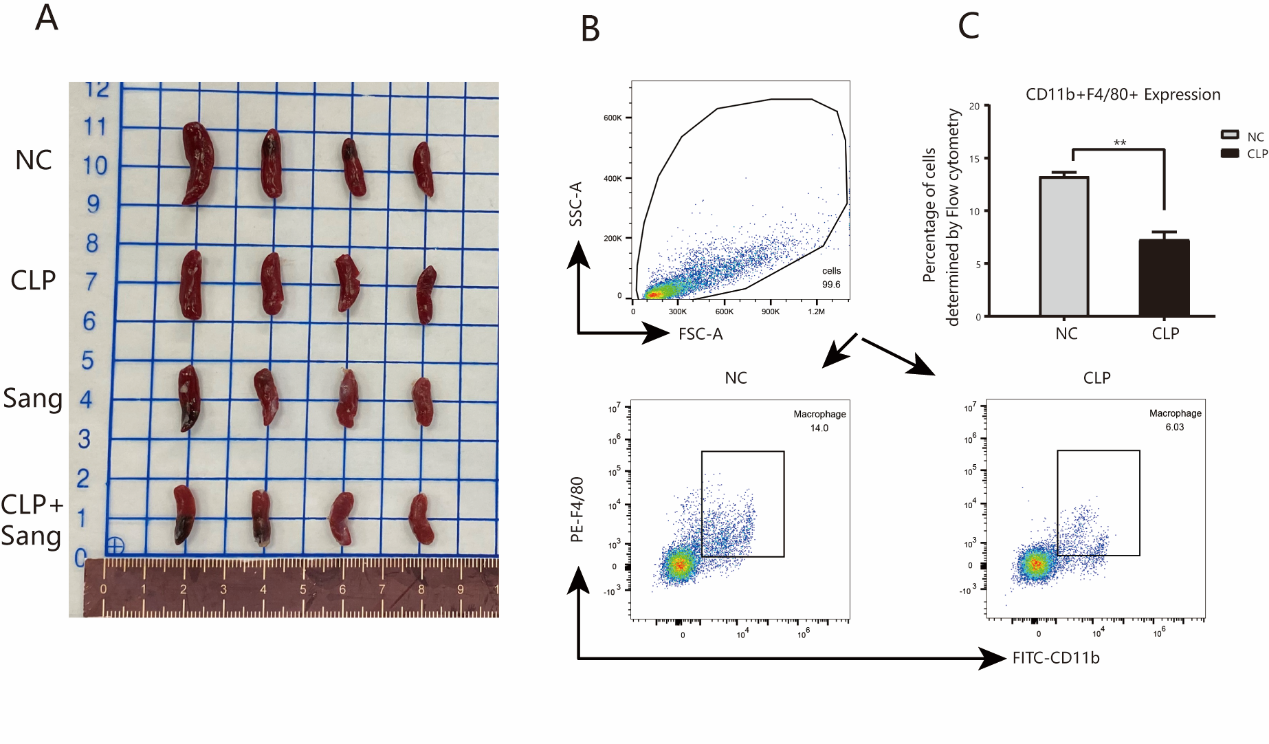


**Supplementary Figure 3.** Clodronate liposome (CLP) treatment depleted macrophages in the spleen.(A) The pictures of spleens from each group. (B) Flow cytometry analysis shows the percentage of CD11b+ F4/80+ macrophages in the spleen of NC and CLP groups. (C) Bar graph shows quantification of the percentage of CD11b+F4/80+macrophages in the spleen of NC and CLP groups. Data are mean ± SD, n=4 per group. **p < 0.01.

**Supplement Table 1**：Antibodies used in this study.

| **Antibodies** | **commercia source information** | **dilutions** |
| --- | --- | --- |
| CD206 | CST(lot:24595) | 1:2000 |
| CD68 | CST(lot: 29176) | 1:2000 |
| CD31 | R&D(lot:YZU0118041) | 1:4000 |
| VEGF | R&D(lot:YZ1417101) | 1:4000 |
| β-Catenin | CST(lot:8480) | 1:1000 |
| WNT5A  GAPDH  Anti-rabbit IgG  Anti-goat IgG | CST(lot:2392)  CST(lot: 2118)  CST(lot:7074)  BOSTER(lot:BA1060); | 1:1000  1:2000~1:4000  1:2000~1:4000  1:2000 |

**Supplement Table 2**：Primers used in this study.

| **Gene** | **Sequence (5’-3’)** |  |
| --- | --- | --- |
| *CD68*-forward | ACTTCGGGCCATGTTTCTCT |  |
| *CD68*-reverse  *CD206*-forward | GCTGGTAGGTTGATTGTCGT  GGAATCAAGGGCACAGAGTTA |  |
| *CD206*-reverse | ATTGTGGAGCAGATGGAA |  |
| *Arg1*-forward | AGGAACTGGCTGAAGTGGTTA |  |
| *Arg1*-reverse | GATGAGAAAGGAAAGTGGCTGT |  |
| *Gapdh*-forward | AAGGAGAGCAGAAGTCCCATGA |  |
| *Gapdh*- reverse | CTCAATCGGACGGCAGTAGCT |  |
| *Wnt1*-F | ATCCATCTCTCCCACCTCCTAC |  |
| *Wnt1*-R | GAATCTTTCTCTCACCCTCTGG | |
| *Wnt2*-F | GTGATGTGTGACAATGTGCCA | |
| *Wnt2*-R | GTTGCAGTTCCAGCGATGC | |
| *Wnt2b*-F | ACCTTCCTCTACCCTCAATCCT | |
| *Wnt2b*-R | TCACTCAGCCTCCTAAATCCAT | |
| *Wnt3*-F | AGCGTAGCAGAAGGTGTGAAG | |
| *Wnt3*-R | CCAGGTGGCCCCTTATGATG | |
| *Wnt3a*-F | TCGGAGATGGTGGTAGAGAAAC | |
| *Wnt3a*-R | TCGCAGAAGTTGGGTGAGG | |
| *Wnt4*-F | AGAACTGGAGAAGTGTGGCTGT | |
| *Wnt4*-R | AAAGGACTGTGAGAAGGCTACG | |
| *Wnt5a*-F | GTCCTTTGAGATGGGTGGTATC | |
| *Wnt5a-*R | ACCTCTGGGTTAGGGAGTGTCT | |
| *Wnt5b*-F | TGTCAGTTGTATCAGGAGCACA | |
| *Wnt5b*-R | GTGAAGGCAGTCTCTCGGCTA | |
| *Wnt6*-F | TTTACACCAGCCCACGAAAG | |
| *Wnt6*-R | ACTCACCCATCCATCCCAGTA | |
| *Wnt7a*-F | GGATGCTCACAGGGAAAGAAC | |
| *Wnt7a*-R | GCAGGAAACCCAGAATACCC | |
| *Wnt7b*-F | TGAAGCTGGAATGTAAGTGTCAC | |
| *Wnt7b*-R | CGCTGCGTTGTACTTCTCCT | |
| *Wnt8a*-F | ACGGTGGAATTGTCCTGAGCATG | |
| *Wnt8a*-R | GATGGCAGCAGAGCGGATGG | |
| *Wnt8b*-F | GTTTGCTTGGGACCGTTG | |
| *Wnt8b*-R | TCCATTTCGGGAGTCATCA | |
| *Wnt9a*-F | ATGGTGTGTCTGGCTCCTG | |
| *Wnt9a*-R | CAGTGGCTTCATTGGTAGTGCT | |
| *Wnt9b*-F | GGGTGTGTGTGGTGACAATCT | |
| *Wnt9b*-R | GGTCCTTGCTTCCTCTCTTG | |
| *Wnt10a*-F | TCCTGTTCTTCCTACTGCTGCT | |
| *Wnt10a*-R | ACGCACACACACCTCCATC | |
| *Wnt10b*-F | CCACTACAGCCCAGAACCTC | |
| *Wnt10b*-R | GGAGAGACCCTTTCAACAACTG | |
| *Wnt11*-F | CCCTGGAAACGAAGTGTAAATG | |
| *Wnt11*-R | AGGTAGCGGGTCTTGAGGTC | |
| *Wnt16*-F | GCTGTAACCTCCTCTGCTGTG | |
| *Wnt16*-R | GTGGACATCGGTCATACTTTCA | |


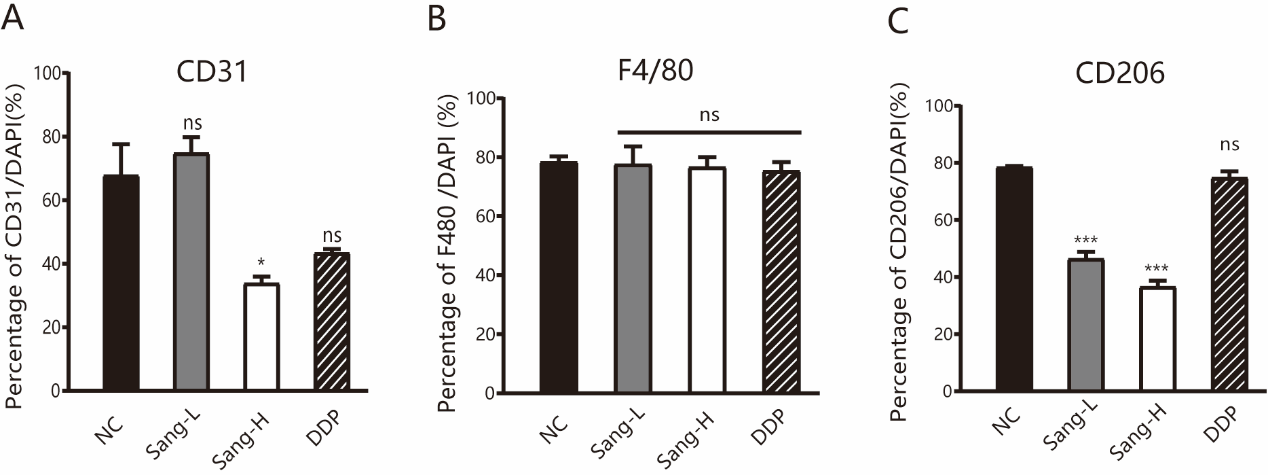


**Supplementary Figure 4:** Quantitative analysis of percentage of (A)CD31+/DAPI cells, (B)F4/80+/DAPI cells, and (C)CD206+/DAPI positive cells in tumor tissue of mouse lung cancer model after treatment with sanguinarine. Data are presented as the mean ± SD of measurements from three photos per group. *, p < 0.05; **, p < 0.01; ***, p < 0.001. ns, not statistically significant (p > 0.05).


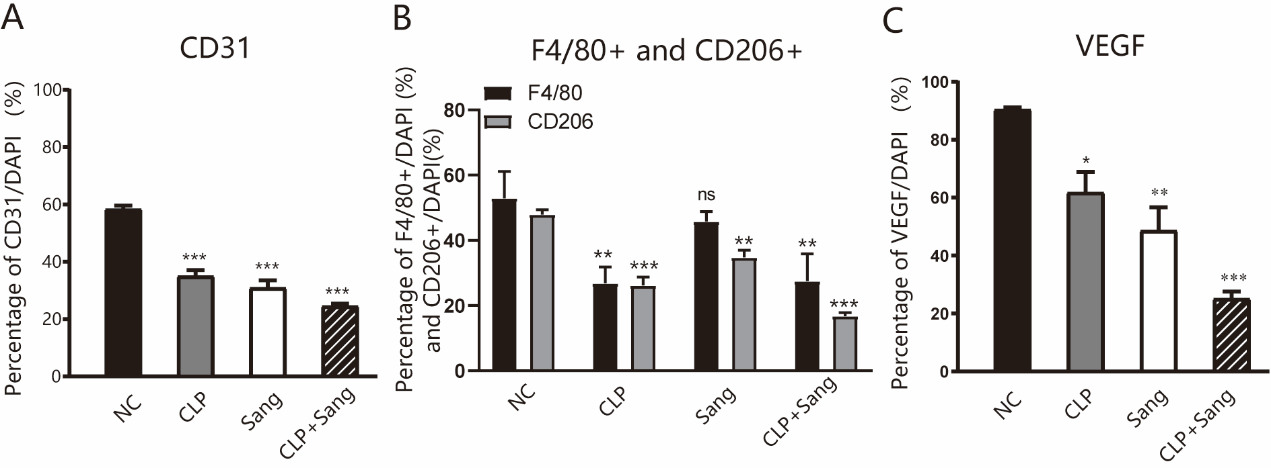


**Supplementary Figure 5:** Quantitative analysis of the percentage of (A)CD31+/DAPI cells, (B) F4/80+/DAPI and CD206/DAPI,and (C)VEGF+/DAPI positive cells in tumor tissue of mouse lung cancer model with macrophage clearance after treatment with sanguinarine. Data are presented as the mean ± SD of measurements from three photos per group. *, p < 0.05; **, p < 0.01; ***, p < 0.001. ns, not statistically significant (p > 0.05).


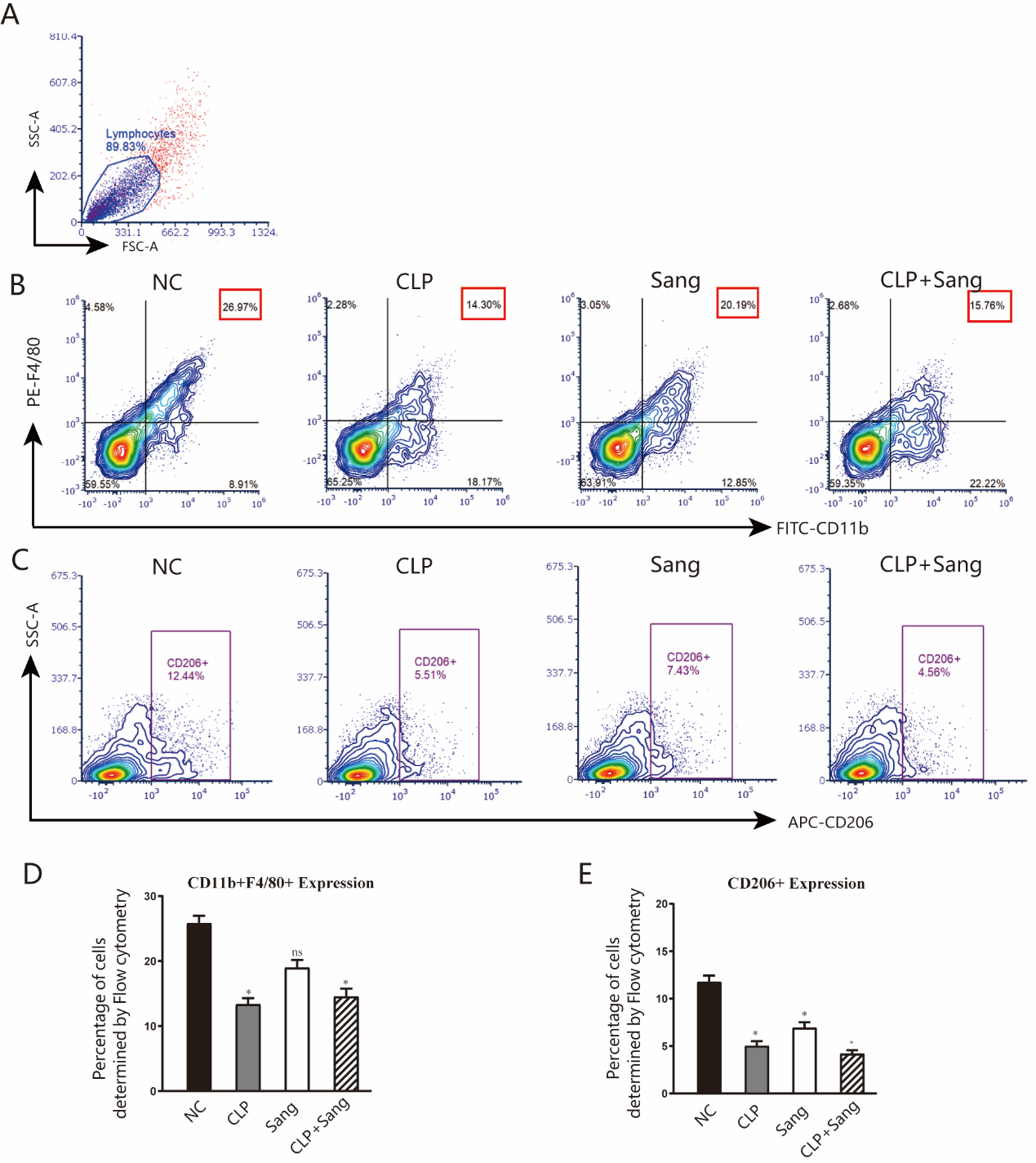


**Supplementary Figure 6:** Single-cell suspension of mouse tumor tissue (n=3) was stained with M0 and M2 antibodies. (A) the strategy of Gating, (B) the total macrophage that infiltrated into the tumor stroma were marked as CD11b+F4/80+, (C) and the M2-type macrophage were stained as CD11b+F4/80+CD206+. The histogram shows the quantitative percentage of (D) CD11b+F4/80 macrophage and (E)CD11b+F4/80+CD206+ in tumor. Data are mean ± SD, n=3 per group. *, p < 0.05; **, p < 0.01; ***, p < 0.001. ns, not statistically significant (p > 0.05).
